# Microfabricated and 3-D Printed Soft Bioelectronic Constructs from PAn-PAAMPSA-Containing Hydrogels

**DOI:** 10.3390/bioengineering5040087

**Published:** 2018-10-17

**Authors:** John R. Aggas, Sara Abasi, Blake Smith, Michael Zimmerman, Michael Deprest, Anthony Guiseppi-Elie

**Affiliations:** 1 Bioelectronics, Biosensors and Biochips (C3B^®^), Department of Biomedical Engineering, Texas A&M University, College Station, TX 77843, USA; jraggas1@tamu.edu (J.R.A.); saraabasi@tamu.edu (S.A.); basmith33@tamu.edu (B.S.); michael_zim@tamu.edu (M.Z.); mdeprest@tamu.edu (M.D.); 2 ABTECH Scientific, Inc., Biotechnology Research Park, 800 East Leigh Street, Richmond, VA 23219, USA

**Keywords:** electroconductive hydrogels, polyaniline, 3-D printing, microfabrication, 4-D hydrogels, electrical impedance spectroscopy, collagen, NIH/3T3, PC-12

## Abstract

The formation of hybrid bioactive and inherently conductive constructs of composites formed from polyaniline-polyacrylamidomethylpropane sulfonic acid (PAn-PAAMPSA) nanomaterials (0.00–10.0 wt%) within poly(2-hydroxy ethyl methacrylate-*co*-N-{Tris(hydroxymethyl)methyl} acrylamide)-*co*-polyethyleneglycol methacrylate) p(HEMA-*co*-HMMA-*co*-PEGMA) hydrogels was made possible using microlithographic fabrication and 3-D printing. Hybrid constructs formed by combining a non-conductive base (0.00 wt% PAn-PAAMPSA) and electroconductive (ECH) (varying wt% PAn-PAAMPSA) hydrogels using these two production techniques were directly compared. Hydrogels were electrically characterized using two-point probe resistivity and electrochemical impedance spectroscopy. Results show that incorporation of >0.10 wt% PAn-PAAMPSA within the base hydrogel matrices was enough to achieve percolation and high conductivity with a membrane resistance (R_M_) of 2140 Ω and 87.9 Ω for base (0.00 wt%) and ECH (10.0 wt%), respectively. UV-vis spectroscopy of electroconductive hydrogels indicated a bandgap of 2.8 eV that was measurable at concentrations of >0.10 wt% PAn-PAAMPSA. Both base and electroconductive hydrogels supported the attachment and growth of NIH/3T3 fibroblast cells. When the base hydrogel was rendered bioactive by the inclusion of collagen (>200 µg/mL), it also supported the attachment, but not the differentiation, of PC-12 neural progenitor cells.

## 1. Introduction

Microlithography and 3-D printing both serve as powerful manufacturing tools for the production of complex, multi-material structures. Recently, both of these techniques have been applied in tandem to engineer complex 3-D biomedical devices for biosensing, tissue engineering, and nerve regeneration [1]. Both methods allow for hydrogels to mimic the natural extracellular microenvironment to allow interaction between the hydrogel and surrounding cells while taking on a designed, complex 3-D structure. Microlithography, a well-established additive and subtractive technique, uses photomasks in order to block or allow UV-light to develop precise 2-D or 3-D structures on a micron-scale [2]. Synthesis of hydrogel structures using lithographic techniques have rendered high resolution (≈1 µm) structures with no limitation of ink viscosity but are limited to photosensitive materials [3]. 3-D printing, which is a purely additive technique, uses extrusion-based layer-by-layer deposition wherein each layer may be UV-cross-linked. While the current state of 3-D bioprinting hydrogels has not yet matched the resolution afforded by photolithographic techniques, it does offer the ability to print larger 3-D structures with moderate resolution (200–1000 µm) [3]. Moreover, 3-D printing offers the potential to produce complex 3-D structures from multiple materials assembled into hybrid architectures.

The creation of bio-nanocomposites that incorporate intrinsically conductive polymers (ICPs), such as polyaniline-poly(2-acrylamido-2-methylpropanesulfonic acid) (PAn-PAAMPSA), into highly-hydrated, 3-D cross-linked hydrogels, such as poly(HEMA-*co*-HMMA-*co*-PEGMA), to create electroconductive hydrogels (ECHs) allows the development of conductive inks that may be microlithographically fabricated or 3-D printed to yield constructs used to support axonal regrowth and tissue innervation [4]. In the presence of a permissive peripheral nerve graft, damaged axons of the peripheral nervous system (PNS) readily regenerate (1.0 mm/day) in large mammals and humans to allow recovery of function. These novel biomaterials can be patterned into customizable nerve guides, conduits, and scaffolds, each of which are personalized to a specific patient, with the promise of accelerated nerve regrowth, in an effort to outperform the “gold standard,” the nerve autograft, by bridging nerve gaps >20 mm at rates greater than the typical 1 mm per day [5].

However, in order to incorporate new rapid prototyping technologies, such as the ability to 3-D print bioactive, electroconductive hydrogels, certain manufacturing challenges must be addressed, similar to the manner in which such challenges were addressed when microlithography was becoming a state-of-the-industry technique in semiconductor manufacturing. Specifically, 3-D printing of protein-rich or cell laden hydrogel bio-inks must: (1) possess rheological properties (viscosity) suitable for accurate 3-D printing, (2) extrude at shear rates that do not adversely impact protein unfolding or cellular behavior (from rupture of cell membranes to sub-cellular activation of shear-sensitive pathways), and (3) result in micro-scale architectures that mimic the extracellular matrix (ECM). To achieve this, improved understanding of the role of and interactions between biological agents, physicochemical properties, and engineering factors is required [6].

This paper reports on the production of three exemplar constructs. The first is a simple hybrid hydrogel disk (ϕ = 10 mm; *l* = 1.0 mm) fashioned as two halves, each comprised of hydrogels of different composition and electrical conductivity. The second is a puzzle piece design, a more complicated hybrid structure, comprised of hybrid hydrogels. The third is the most complex construct comprised of a hydrogel disk (ϕ = 10 mm; *l* = 1.0 mm) possessing a pentagonal arrangement of five evenly spaced cylindrical cavities (ϕ = 1.25 mm; *l* = 1.0 mm), each filled with electroconductive hydrogels of increasing amounts of PAn-PAAMPSA (0.00–1.00 wt%). The disks were produced using both microlithographic fabrication and 3-D printing techniques. The conditions of fabrication and the resulting disks created using the two methods were directly compared. Furthermore, the electrical and mechanical properties of the ECHs were explored. Finally, NIH/3T3 cells were cultured on both non-conductive, base (0.00 wt% PAn-PAAMPSA) and electroconductive (0.10 wt% PAn-PAAMPSA) hydrogels and their growth and morphology were assessed using fluorescence microscopy. PC-12 cells were cultured on the non-conductive base (0.00 wt% PAn-PAAMPSA) rendered bioactive by the inclusion of collagen.

## 2. Materials and Methods

### 2.1. Materials

The base hydrogel was formulated from mixtures of the monomers, 2-hydroxyethylmethacrylate (HEMA) (Sigma-Aldrich, Saint Louis, MO, USA) (84.5 mol%), N-{Tris(hydroxymethyl)methyl} acrylamide (HMMA) (Sigma-Aldrich) (5.0 mol%), and poly(ethylene glycol) monomethacrylate (PEG(360)MA) (Polysciences, Warrington, PA, USA) (5.0 mol%). Additionally, it contained the crosslinker, tetra(ethylene glycol)diacrylate (TEGDA) (3.0 mol%), a pre-polymer to control viscosity, poly(N-vinylpyrrolidone) (pNVP) (MW = 1.3M) (2.0 mol% of repeat units), and the photoinitiator, 2,2-dimethoxy-2-phenylacetophenone (DMPA) (0.5 mol%), all from Sigma-Aldrich (St. Louis, MO, USA). In formulations used in cell culture studies, pNVP was replaced by poly(HEMA) (MW = 1.0 × 10^6^). Electroconductive hydrogels were synthesized from the base hydrogel formulation (preceding) by the addition of separately synthesized polyaniline-poly(2-acrylamido-2-methylpropanesulfonic acid) (PAn-PAAMPSA) nanomaterials [7]. Base and electroconductive hydrogels were rendered bioactive by the addition of collagen type I (calf skin, Sigma-Aldrich). NIH/3T3 (*Mus musculus*, mouse, embryo) and PC-12 cell (*Rattus norvegicus*, rat, adrenal gland) were purchased from American Type Culture Collection (ATCC) (Manassas, VA, USA) and cultured according to standard protocols. Cell culture materials: RPMI-1640 Medium (With L-glutamine and sodium bicarbonate), Dulbecco’s Modified Eagle’s Medium (DMEM), fetal bovine serum (FBS), horse serum, Trypsin, and Penicillin-Streptomycin (10,000 units penicillin and 10 mg streptomycin/mL, PS) were purchased from Sigma-Aldrich. Rhodamine-Phalloidin and 4′,6-diamidino-2-phenylindole (DAPI) were purchased from Thermo-Scientific (Rockford, IL, USA). Phosphate-buffered Saline (PBS) and 4-(2-hydroxyethyl)-1-piperazineethanesulfonic acid (HEPES) buffer were prepared by dissolving a PBS tablet (Gibco, Thermo Fisher Scientific, Waltham, MA, USA) and HEPES (Sigma, St. Louis, MO, USA) in DI water to yield a 0.1 M and 25 mM buffers, respectively, with a pH adjusted to 7.35–7.45.

### 2.2. Methods

#### 2.2.1. Microlithographic Fabrication

**Figure 1:**
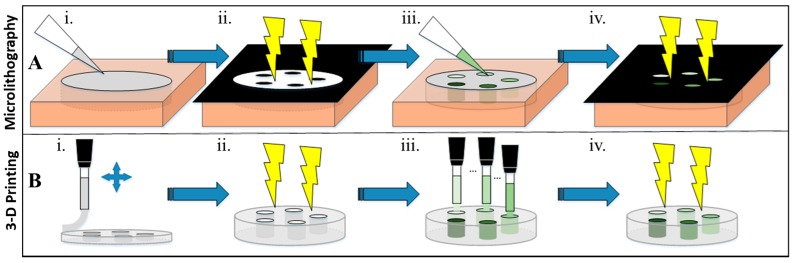
Scheme of microlithographically produced and 3-D printed hybrid hydrogel structures. (**A**) Microlithography: (i) 50 µL of hydrogel cocktail was pipetted into a silicone isolator and then (ii) covered with a mask and crosslinked for 5 min under UV. (iii) The mask was removed, the non-crosslinked cocktail was washed off, and electroconductive hydrogels were pipetted into the wells. (iv) The wells were then covered in a reverse mask and crosslinked for 5 min under UV. (**B**) 3-D Printing: (i) A syringe loaded with a hydrogel cocktail was used to extrusion print the outer part of the hydrogel (layer-by-layer) with a (ii) 30 s UV crosslinking done between each layer. (iii) The five wells were then printed with the electroconductive cocktails and subsequently (iv) UV crosslinked for 5 min.

Photopatterned hydrogels were fashioned by use of a negative photomask. Several masks were produced by printing desired patterns onto acetate transparencies at 2400 dpi, ranging from simple half circles, to intricate puzzle-piece designs. The final photomask produced a hydrogel disk with 5 pentagonally arranged wells. The process of fabrication is shown in Figure 1A. The base, a non-conductive hydrogel, which made the bulk of the disk, was pipetted into a cylindrical silicone mold (621101 Grace Biolabs φ = 10.0 mm × *l* = 1.0 mm depth). The non-masked parts of the molded hydrogel were exposed for 5 min to UV within a CX-2000 UV Crosslinker (UVP, LLC, Upland, CA, USA) at 100 µJ/cm^2^ to initiate polymerization. Non-crosslinked hydrogel under the masks were then washed away with 50/50 *v/v* EtOH/water. Electroconductive hydrogels containing 0.00, 0.01, 0.10, 0.50, and 1.00 wt% PAn-PAAMPSA were pipetted into the non-developed (open) areas and covered with the aligned reverse mask and cross-linked by UV exposure. Following cross-linking, hydrogels were hydrated for successive periods of 1 h in EtOH/HEPES with solution ratios of 100/0, 75/25, 50/50, and 25/75, and terminally stored in a HEPES buffer (pH = 7.4).

#### 2.2.2. 3-D Printing

Hydrogels were rendered 3-D printable by increasing the concentration of pNVP (4, 6, 8, and 12 mol%). For initial studies, only 0.1 wt% PAn-PAAMPSA was incorporated into the printable, electroconductive formulation. Electroconductive hydrogel inks were placed in 30 cc syringes with a 200-micron needle inner diameter. 3-D printing was done using an EnvisionTEC 3-D Bioplotter and accompanying software, Perfactory RP, and Bioplotter RP (EnvisionTEC GmbH, Gladbeck, Germany). The EnvisionTEC 3-D Bioplotter was housed within a custom-fabricated biosafety cabinet for BSL-2 printing of human cells. Hydrogels were printed at a z-offset of 0.12 mm, a printing speed of 8.0 mm/s, and an extrusion pressure of 0.8 bar. The process of fabrication is shown in Figure 1B. The 3-D constructs were printed layer-by-layer, with UV light exposures for 30 s between layers. Following crosslinking, hydrogels were hydrated using the same protocol used for photopatterned hydrogels.

### 2.3. Characterization of Hydrogels

#### 2.3.1. Rheological and Optical Characterization of Hydrogel Cocktails

The complex viscosity of 0.00 and 0.10 wt% PAn-PAAMPSA hydrogel cocktails formulated for microlithography and 3-D printing were measured using an Anton Paar MCR 301 Rheometer (Anton Paar GmbH, Graz, Austria) using a parallel plate geometry with a 50 mm stainless steel plate, gap width of 0.5 mm, and a 20 °C working temperature. Complex viscosities were measured from 0.1 to 100 rad/s with a strain of 1%. UV-Vis spectra of 0.00, 0.10, 0.50, and 1.0 wt% PAn-PAAMPSA hydrogel cocktails (before crosslinking) formulated for microlithography and 3-D printing were recorded using a BioTek PowerWave XS microplate photometer (BioTek, Winooski, VT, USA) with a spectrum wave number range from 300–900 nm.

#### 2.3.2. Electrical and Electrochemical Characterization

Alternating current (AC) electrical impedance spectroscopy (EIS) measurements of electroconductive hydrogels synthesized to contain 0.00, 0.10, 1.00, and 10.0 wt% PAn-PAAMPSA fabricated for both microlithographic and 3-D printing techniques were made at room temperature using a 10 mV peak-to-peak interrogating sine wave over the range 10 mHz–1.0 MHz using a Versastat 4 (Princeton Applied Research, Oak Ridge, TN, USA). Hydrogel constructs were cast into cylindrical silicone molds, UV-crosslinked, hydrated, and set between two opposing gold planar metal electrodes (PME-Au; ABTECH Scientific, Inc., Richmond, VA, USA). Hydrogels were fixtured between opposing PME-Au electrodes to ensure complete electrical contact between the hydrogels and the PMEs and reproducibility for all EIS measurements. Equivalent circuit analysis of acquired impedance spectra was completed using ZSimpWin (v3.60, Princeton Applied Research, Oak Ridge, TN, USA). The two-point direct current (DC) resistance of UV-cured, electroconductive hydrogels synthesized to contain 0.00, 0.10, 0.50, and 1.00 wt% PAn-PAAMPSA fabricated by both microlithographic and 3-D printing techniques was measured using a Multimeter (Model 2010, Keithley, Cleveland, OH, USA). Five replicate measurements were made by keeping a constant distance between the probe tips and pressure on the hydrogels.

#### 2.3.3. Morphological Characterization of Electroconductive Hydrogels

Surface morphology of samples were imaged using scanning electron microscopy (SEM) utilizing a JEOL JSM-7500F FE-SEM (JEOL, Ltd., Akishima, Tokyo, Japan). Both microlithographically produced and 3-D printed hydrogels with the inclusion of 0.00, 0.10, and 1.00 wt% PAn-PAAMPSA were imaged. Hydrogels were synthesized and hydrated via the steps above. After hydration, hydrogels were placed in liquid nitrogen for 30 s and freeze-fractured. Hydrogels were then dried in a Turbo-Vap V500 (Caliper LifeSciences, Hopkinton, MA, USA) solvent evaporation system for 48 h at 37 °C before imaging.

### 2.4. Cell Culture Study

In order to study the suitability of the hydrogels for supporting cell growth, the base (0.00 wt% PAn-PAAMPSA) and one composition of electroconductive hydrogel (0.10 wt% PAn-PAAMPSA) were synthesized and prepared as described in Section 2.2.1. Following sequential gradual hydration/extraction in decreasing concentrations of ethanol in cell culture grade PBS, samples were sterilized with 30 min germicidal UV irradiation. Prior to cell culture, the hydrogels were incubated in DMEM containing FBS for 2 h in a 5% CO_2_ incubator. NIH/3T3 cells were cultured according to the standard protocol and seeded on the hydrogels at a density of 3 × 10^5^ cells/mL. The attachment and growth of the cells were assessed after 24 h. The elongation of the cells was calculated from the ratio of length and width of the cells using ImageJ 1.52a (Freeware, National Institutes of Health, Bethesda, MD, USA) freeware and represented as an aspect ratio. The optical properties of the electroconductive hydrogel do not readily allow visualization of cells with bright field microscopy, hence cells were fluorescently tagged. Cell nuclei and F-actin filaments were fluorescently stained with DAPI and Rhodamine phalloidin, respectively. The cells were fixed and stained according to well stablished protocols [8] and visualized using fluorescent microscopy. For culturing PC-12 cells, the base hydrogel with 2.0% pNVP was prepared and rendered bioactive via the addition of collagen type I (calf skin, Sigma-Aldrich). Different concentrations of collagen (10, 50, 100, 200, and 500 µg/mL) were evaluated. Bioactive hydrogel disks were prepared via UV-crosslinking the cocktail containing collagen for 5 min at 100 µJ/cm^2^. The isolated disks were hydrated in cell culture grade PBS for 48 h. A ninhydrin (Sigma-Aldrich) assay was used to confirm that the collagen was retained within the hydrogel and did not leach out during hydration. Hydrogel disks were placed in 24-well Falcon Polystyrene Microplates (353226 Corning Inc., Corning, NY, USA) and seeded with PC-12 cells (≈3 × 10^5^/mL). Twenty-four hours after seeding, the hydrogel disks were gently washed with PBS to remove unattached cells and the attached cells were visualized using an inverted Axio Vert A1 microscope (Zeiss, Oberkochen, Germany).

## 3. Results and Discussion

### 3.1. Microfabricated and 3-D Printed Hydrogels

**Figure 2:**
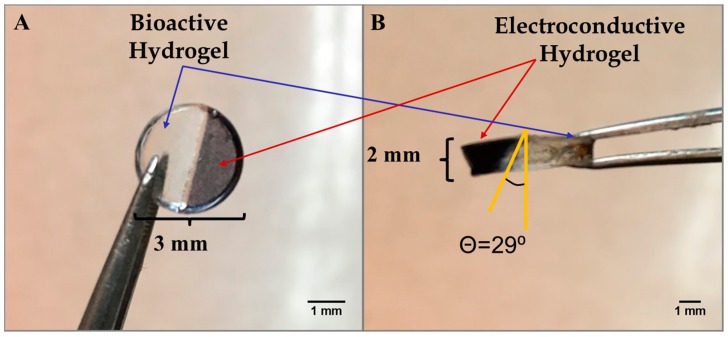
Microlithographic fabrication of a hybrid construct based on the use of photomasks to create adjacent non-conductive p(HEMA-*co*-HMMA-*co*-PEGMA) hydrogels and electroconductive (1.00 wt% PAn-PAAMPSA) hydrogels. Hybrid hydrogel disk (**A**) top view and (**B**) side view.

**Figure 3:**
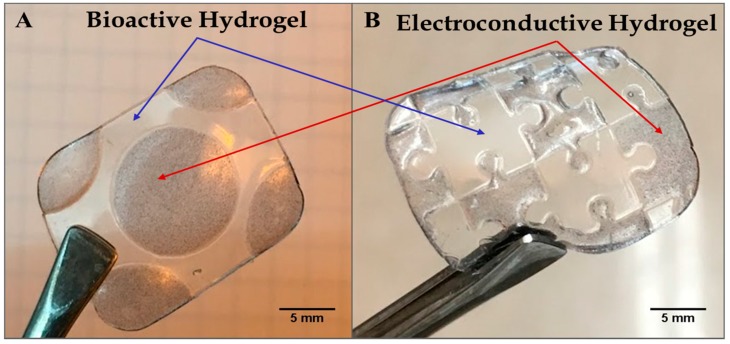
Microlithographic fabrication of hybrid constructs based on the use of photomasks to create complex structures of adjacent non-conductive p(HEMA-*co*-HMMA-*co*-PEGMA) hydrogels and electroconductive (1.00 wt% PAn-PAAMPSA) hydrogels. (**A**) Circular electroconductive hydrogel surrounded by non-conductive hydrogel and (**B**) puzzle-piece pattern of electroconductive and non-conductive, bioactive hydrogel.

Figure 2A,B shows a hybrid hydrogel disk fashioned using the photopatterning technique, wherein one half of the hydrogel disk was produced from the bioactive hydrogel component and the other half incorporated from the electroconductive PAn-PAAMPSA (0.10 wt%) component. The disk was created with a 3 mm diameter and 2 mm thickness. The non-conductive (clear) half of the disk was crosslinked first, which brings into view two main limitations seen when attempting to create hydrogel structures via lithography: (1) the distance between the photomask and the hydrogel (i.e., direct contact or proximity contact); and (2) unintended crosslinking of hydrogel located under the mask via diffusion of free radicals that influences acuity [9]. In this particular case, free radicals created in the non-masked area diffused outward leading to non-desired cross-linking and loss of resolution [10]. Hydrogels consisting of a single layer produced via the mask technique have been shown to retain high resolution only up to ≈1 mm, while thicker constructs can be created via the use of other lithographic techniques such as multi-layer patterning or stereolithography [11,12]. For this reason, all other hydrogels were made with a thickness of ≤1 mm. Figure 3A,B shows complex hybrid hydrogel structures created using the photopatterning technique with both the bioactive and electroconductive components. The puzzle-piece hybrid structure was able to accurately reproduce non-trivial features as low as 1.0 mm with a hydrogel thickness of 1.0 mm.

**Figure 4:**
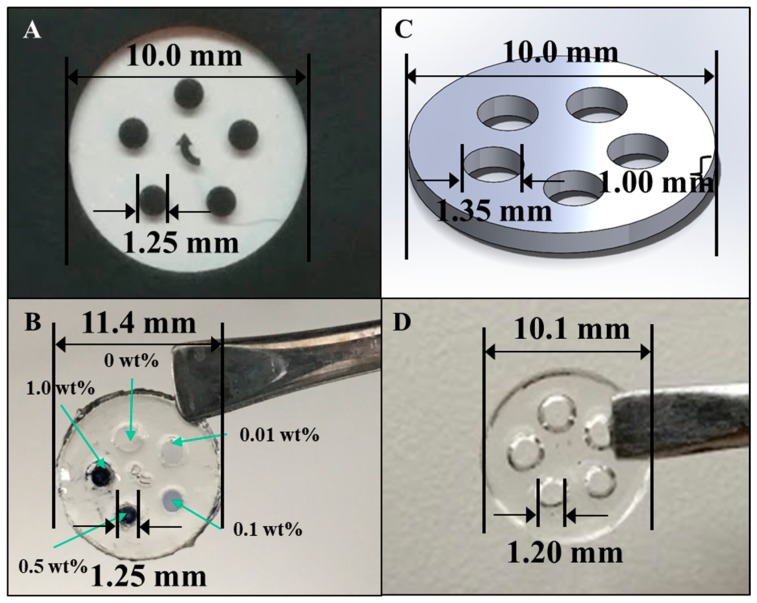
(**A**) Photomask used in the microfabrication technique and (**B**) the resultant hydrogel array with array elements of 0.00, 0.01, 0.01, 0.50, and 1.00 wt% PAn-PAAMPSA. (**C**) Solidworks^®^ part used in the 3-D printing technique and (**D**) the resultant hydrogel array.

A photomask was prepared for use in the microlithographic fabrication of an electroconductive hydrogel array and is shown in Figure 4A. The aim was to fabricate an array of cylindrical features, each array element being ϕ = 1.25 mm and *l* = 1.0 mm, that connected the two surfaces of the larger hosting, bioactive hydrogel disk. The larger hosting disk (φ = 10.0 mm × *l* = 1.0 mm) was to be fashioned from a bioactive, protein-containing, p(HEMA-*co*-HMMA-*co*-PEGMA) hydrogel and each array element was to be fashioned from a different composition of PAn-PAAMPSA containing electroconductive hydrogel. A completed, microfabricated hydrogel with 0.00, 0.01, 0.10, 0.50, and 1.00 wt% PAn-PAAMPSA ECH is shown in Figure 4B. Similarly, the Solidworks^®^ (Dassault Systèmes SolidWorks Corp., Waltham, MA, USA) part file used to render the 3-D printed hydrogel, shown in Figure 4C, was designed with an outer diameter (φ = 10.0 mm × *l* = 1.0 mm), inner circle diameter of ϕ = 1.35 mm, and *l* = 1.0 mm. The inner diameter of the hydrogel wells was made 0.1 mm larger than those of the structures produced via lithography due to diffusion of the hydrogel after it has been extruded [13]. The 3-D printed outer, non-conductive hydrogel is shown in Figure 4D.

A comparison of the hydrogel structures in Figure 4B,D revealed that both methods were viable for constructing the complex disk shape. While the microfabricated structure remained more true to the desired dimensions of the inner circle (microfabricated: <1% error, 3-D print: 11% error), the 3-D printed hydrogel remained more true to dimensions of the outer circle (microfabricated: 14% error, 3-D print: 1% error).

To enable 3-D printing by extrusion, the rheology (viscosity) of hydrogels is one of the most important factors [13]. A hydrogel that is too viscous will not extrude through a needle tip at available hydrostatic pressures, while a hydrogel with too low a viscosity will flow directly through the needle tip in an uncontrolled manner and print a deformed structure [14]. Moreover, flow of the extruded layers following printing, but prior to UV-crosslinking, can affect resolution. In addition, the need to parameterize each new material used to define the proper print speed, hydrostatic pressure, and appropriate needle diameter is time-consuming up front. However, as long as the same material is being used, there is no need to change these parameters. The 3-D printing technique does offer high throughput, as multiple hydrogel compositions can be printed in the same job. Intricate constructs, such as the hybrid, jigsaw-puzzle piece shown in Figure 3B, are more easily approached via the microlithographic technique. Non-uniform shapes, such as the jig-saw puzzle, present a challenge to infill with the available, linear, or space-filling pattern techniques of 3-D printing, especially when a solid hydrogel is desired (100% infill).

Both microfabricated and 3-D printed hydrogels show promise for use in soft electronics, bioelectronics, and regenerative engineering. Specifically, fabrication of hybrid hydrogels with multiple bioinks to achieve novel materials and properties can be integrated into implantable scaffolds for nerve regeneration, where incorporation of nerve growth factors can lead to increased nerve penetration and cellular infiltration resulting in a quicker rate of regeneration [15]. In addition, increasingly complex designs for bioelectronics are being used to produce responsive actuators, organic field effect transistors (OFET), and organic light emitting diodes (OLED), each of which can be produced via photopatterning and 3-D printing techniques [16].

### 3.2. Rheological Characteristics of Hydrogel Cocktails

Viscoelastic properties of hydrogels, while not an important feature when creating constructs via microlithography, is one of the most important parameters in 3-D printing. Complex viscosity (|η*|), is a function of the storage modulus (G′), loss modulus (G″), and angular frequency (ω) (Equation (1)).

(1) $$\;\left|\eta^{*}\right|=\sqrt{{\left(\frac{G^{\prime \prime }}{\omega }\right)}^{2}+{\left(\frac{G^{\prime }}{\omega }\right)}^{2}}\;$$

**Figure 5:**
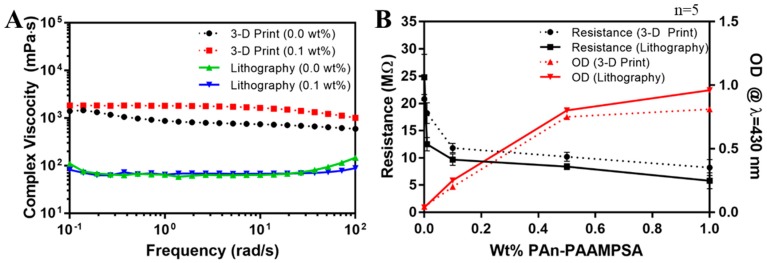
(**A**) Complex viscosities of hydrogel cocktails formulated for microlithography (2 mol% pNVP) and 3-D printing (12 mol% pNVP) and containing 0.00 and 0.10 wt% PAn-PAAMPSA. (**B**) Two-point DC resistance (MΩ) and optical density (OD) at λ = 430 nm of p(HEMA-*co*-HMMA-*co*-PEGMA) hydrogels containing at 0.00, 0.01, 0.10, 0.50, and 1.00 wt% PAn-PAAMPSA.

Figure 5A shows complex viscosities of hydrogel cocktails formulated for microlithography (2 mol% pNVP) and 3-D printing (12 mol% pNVP) with 0.00 and 0.10 wt% PAn-PAAMPSA. Complex viscosity measurements made as a function of angular frequency could be equated to shear viscosity versus shear rate for hydrogels by applying the Cox–Merz rule [17]. Incorporation of 0.10 wt% PAn-PAAMPSA had no significant effect on viscosity. The increase in pNVP, from 2 mol% to 12 mol%, was required to render the cocktail with |η*| > 300 mPa·s, the minimum viscosity required for 3-D printing mechanically stable structures [18], and clearly had a dramatic effect on the formulated cocktail viscosity.

### 3.3. Optical, Electrical, and Electrochemical Characterization

Figure 5B shows two-point probe resistance values (black) of hydrogels and optical density (OD) (red) at λ = 430 nm of hydrogel cocktails measured as a function of wt% PAn-PAAMPSA (0.00, 0.01, 0.10, 0.50, and 1.00 wt%) produced using microlithography (solid lines) and 3-D printing (dashed lines). The resistance dropped significantly between 0.00 and 0.10 wt% PAn-PAAMPSA regardless of hydrogel formulation. At >0.10 wt% PAn-PAAMPSA percolation was reached as indicated by the drop-in resistance and the increase in OD that produced a bandgap energy of ≈2.8 eV in Tauc plots (not shown). The OD at λ = 430 nm was discernable at 0.10 wt% PAn-PAAMPSA, indicative of polaron transitions [19,20].

**Figure 6:**
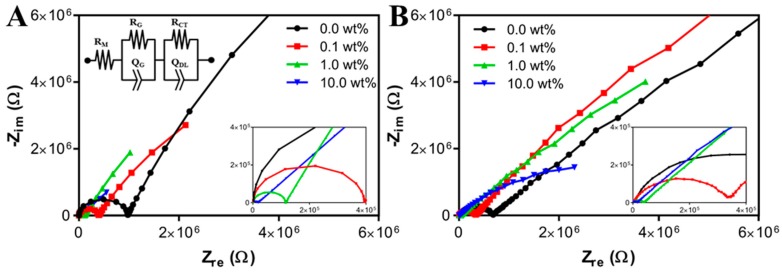
Nyquist plots of electroconductive hydrogels (ECH) containing 0.0, 0.1, 1.0, and 10 wt% PAn-PAAMPSA measured at room temperature (RT) in air from 10 mHz to 1.0 MHz using a 10 mV peak-to-peak sine wave. (**A**) Hydrogels formulated for microlithography containing 2 mol% pNVP and the equivalent R(QR)(QR) circuit (inset). (**B**) Hydrogels formulated for 3-D printing containing 12 mol% pNVP.

Figure 6 shows Nyquist plots (Z*_real_* vs. *Z_imag_*) of ECH’s with 0.00, 0.10, 0.50, and 10.0 wt% PAn-PAAMPSA created via microlithography (A) and 3-D printing (B). Impedance data was fitted to the equivalent circuit, R(QR)(QR), where the first series resistance, R_M_, was attributed to the membrane resistance. The Q, also known as a constant phase element (CPE), modelled an imperfect capacitor. The first parallel QR was attributed to geometrical resistance, R_G_, and geometrical capacitance, Q_G_, of the interrogation electrodes, which became apparent at high frequencies [21]. The larger semicircles of the Nyquist plots were attributed to the second parallel QR, and were attributed to charge transfer resistance, R_CT_, and double layer capacitance, Q_DL_, of the electrified electrode–polymer interface. Most notably, the membrane resistance decreased from 2140 Ω to 87.9 Ω as composition was changed from 0.00 wt% to 10.0 wt% PAn-PAAMPSA within the microlithographically produced hydrogels. Similarly, 3-D printed hydrogels showed a decrease from 3310 Ω to 303 Ω. The differences in the extracted membrane resistances likely arose from the increase in pNVP content required to enable extrusion printing of the 3-D printed constructs. The extracted equivalent circuit values are shown in Table 1.

The inclusion of the intrinsically conductive polymer, PAn-PAAMPSA, within p(HEMA-*co*-HMMA-*co*-PEGMA) hydrogel matrices served to increase the conductivity of the bulk hydrogel as it transitioned from ionic conduction to polaronic conduction. Such hydrogel nanocomposites, being tunable in their electrical properties, have the potential to serve as the medium of charge conduction of bioelectrical signals in vivo [22] in applications such as conduits in nerve regeneration and in cardiac patch synchronization. Furthermore, the inherently conductive polymer, which exhibits poor mechanical properties, will become compromised when implanted if not fashioned as a component inside a more mechanically stable p(HEMA-*co*-HMMA-*co*-PEGMA) hydrogel matrix [23]. Thus, the inclusion of PAn-PAAMPSA at varying wt% showed similar membrane impedance characteristics for both microlithographically fabricated and 3-D printed hydrogels. The slight changes in polymer composition (increased mol% of pNVP), required for 3-D printed hydrogels, did not result in any significant changes to the membrane impedance.

### 3.4. Morpholicial Characterization of Electroconductive Hydrogels

**Figure 7:**
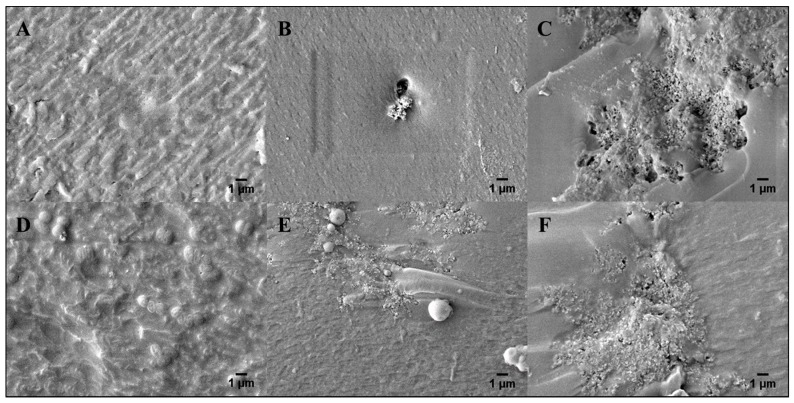
SEM images of freeze-fractured surfaces of microlithographically produced hydrogels with (**A**) 0.00, (**B**) 0.10, and (**C**) 1.00 wt% PAn-PAAMPSA. 3-D printed hydrogels with (**D**) 0.00, (**E**) 0.10, and (**F**) 1.00 wt% PAn-PAAMPSA (magnification: ×5000).

Figure 7 shows SEM images (×5000 magnification) of microlithographically produced (A,B,C) and 3-D printed (D,E,F) hydrogels that contained 0.00, 0.10, and 1.0 wt% PAn-PAAMPSA, respectively. Upon visual inspection, there was no difference in morphology between these two fabrication techniques. Incorporation of PAn-PAAMPSA at as little as 0.10 wt% PAn-PAAMPSA resulted in visible aggregates of the conductive polymer (high levels of aggregation can be avoided by including surfactants such as Pluronic 127 in the hydrogel cocktail), while incorporation of 1.00 wt% PAn-PAAMPSA showed patterns of interconnected aggregates dispersed within the crosslinked hydrogel. Surface roughness of images calculated via ImageJ resulted in higher values for 3-D printed hydrogels (0.00 wt%: 31.32, 0.10 wt%: 30.38, 1.00 wt%: 36.29) when compared to microlithographically produced hydrogels (0.00 wt%: 23.87, 0.10 wt%: 21.98, 1.00 wt%: 27.45).

### 3.5. NIH/3T3 and PC-12 Cells on Hydrogels

**Figure 8:**
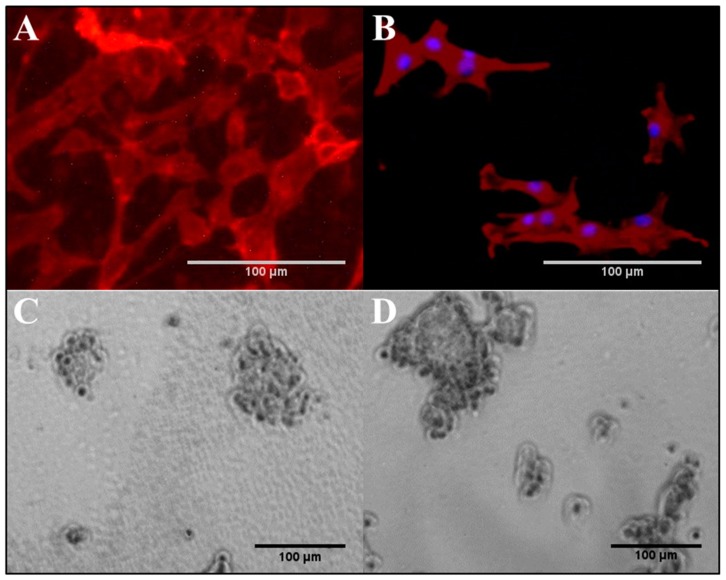
Attachment and growth of cells on p(HEMA-*co-*HMMA-*co*-PEGMA) hydrogels. NIH/3T3 cells on (**A**) non-conductive, base (0.0 wt% PAn-PAAMPSA) and (**B**) electroconductive (0.1 wt% PAn-PAAMPSA). PC-12 cells on non-conductive, base (0.0 wt% PAn-PAAMPSA) with (**C**) 200 µg/mL collagen and (**D**) 500 µg/mL collagen.

According to the results shown in Figure 8A,B, the attachment and growth of NIH/3T3 cells was well supported on both non-conductive, base (0.00 wt% PAn-PAAMPSA), and electroconductive (0.10 wt% PAn-PAAMPSA) hydrogels. Although the hydrogels did not contain any bioactive reagent, such as protein or peptide to support receptor-mediated attachment, the fibroblasts could still attach to these materials and grow. This attachment likely followed the adsorption of proteins secreted by the cells and from the FBS-rich media. Cells established their usual spindle-like morphology but did not elongate to the same extent found on rigid polystyrene plates. The average NIH/3T3 aspect ratio of the cells were 1.57 ± 0.45 and 2.23 ± 1.21 on hydrogel and polystyrene cell culture plates, respectively. The observed limited elongation of the cells is in agreement with previous studies that reported that elongation of fibroblast depends on the modulus of the surface and is limited on soft materials [24]. The data also indicated that the elongation of cells was less on ECH. Fibroblast elongation is governed by the contact guidance on the surface [25], however, its relationship with electrical conductivity of the substrate is not yet fully understood.

PC-12 cells were cultured on p(HEMA-*co*-HMMA-*co*-PEGMA) hydrogels in the same fashion as NIH/3T3 cells; however, the cells did not attach to the hydrogels. PC-12 cells are known to require the presence of bioactive sites, whether in the form of peptide, e.g., RGD, or protein, e.g., collagen, to mediate their attachment, and do not attach to unmodified cell culture plates [8]. Hence the hydrogel was rendered bioactive via the inclusion of 10, 100, 200, and 500 µg/mL collagen. The attachment of PC-12 cells was limited on hydrogels containing less than 200 µg/mL collagen, which indicated that at those concentrations, motifs that supported receptor-mediated attachment did not present on the surface of the gel at adequate density and were not bioavailable to the cells. Figure 8C,D show the bright field micrograph of PC-12 cells attached to the collagen-containing p(HEMA-*co*-HMMA-*co*-PEGMA) hydrogel (200 and 500 µg/mL collagen). The cells maintained their regular chromaffin morphology without any sign of neurite outgrowth.

Fibroblasts are anchorage-dependent cells and must attach to a surface to survive and grow; however, they do not require a surface with a pre-coat layer of protein for attachment as they secrete their own proteins. Moreover, the inclusion of FBS in the media assured an adequate concentration of proteins for adsorption onto the hydrogel. On the other hand, PC-12 cells did require the presence of collagen to attach to the hydrogels. The results indicated the importance of the cell type of interest when developing biomaterials and the need to evaluate with multiple cell lines and possibly, with co-cultures. In the case of nerve conduit development, hydrogels are a good candidate because of their high water content, tunable properties, potential for functionalization and structural similarity to the ECM [26]. However, due to a lack of bioactive sites, they must be synthesized to include proteins and peptides for promoting cell attachment. Here the results suggest that the p(HEMA-*co*-HMMA-*co*-PEGMA) hydrogel prepared with the microfabrication technique is permissive for neuronal progenitor cells.

## 4. Conclusions

Complex, hybrid hydrogel structures can be fabricated using microlithography and 3-D printing techniques for soft bioelectronics. Both techniques can be exploited in the development of complex, multi-material hydrogel structures that show promise for the fabrication of structures with dimensions suitable in the area of nerve regeneration conduits that are aimed to increase the rate and efficiency of peripheral nerve regeneration. Electrical activity can be conferred into hydrogels by as little as 0.1 wt% PAn-PAAMPSA. Initial studies of NIH/3T3 cells on p(HEMA-*co*-HMMA-*co*-PEGMA) indicate that the ECH can adequately support attachment and grow of fibroblasts. On the other hand, PC-12 cells were attached to p(HEMA-*co*-HMMA-*co*-PEGMA) hydrogels only when the hydrogel was rendered bioactive by the inclusion of relatively high concentrations of collagen (>200 µg/mL) within the cocktail.

## Figures and Tables

**Table 1:** Equivalent circuit parameters extracted from impedance spectra shown in Figure 6.

		Microlithography Formulation				3-D Printing Formulation			
	Circuit Parameter	0.0	0.10	1.00	10.0	0.0	0.10	1.00	10.0
Membrane	R_M_ (Ω)	2.14 × 10^3^	1.27 × 10^3^	1.05 × 10^3^	8.79 × 10^1^	3.31 × 10^3^	1.83 × 10^3^	9.23 × 10^2^	3.03 × 10^2^
Electrode Geometry	Q_G_ (S·s^n^)	1.62 × 10^−11^	2.02 × 10^−11^	3.44 × 10^−11^	2.73 × 10^−9^	3.34 × 10^−11^	4.44 × 10^−10^	3.95 × 10^−10^	4.22 × 10^−10^
	n (0 < n < 1)	1.00 × 10^0^	1.00 × 10^0^	1.00 × 10^0^	7.48 × 10^−1^	8.38 × 10^−1^	8.11 × 10^−1^	8.25 × 10^−1^	8.45 × 10^−1^
	R_G_ (Ω)	9.72 × 10^5^	3.92 × 10^5^	1.14 × 10^5^	1.85 × 10^4^	6.64 × 10^5^	9.49 × 10^4^	3.59 × 10^4^	1.58 × 10^4^
Electrode Interface	Q_DL_ (S·s^n^)	1.06 × 10^−6^	2.23 × 10^−6^	3.79 × 10^−6^	5.76 × 10^−6^	3.88 × 10^−7^	4.26 × 10^−7^	8.61 × 10^−7^	1.29 × 10^−6^
	n (0 < n <1)	7.97 × 10^−1^	7.45 × 10^−1^	7.59 × 10^−1^	5.84 × 10^−1^	5.75 × 10^−1^	6.69 × 10^−1^	5.89 × 10^−1^	6.09 × 10^−1^
	R_CT_ (Ω)	5.47 × 10^7^	2.97 × 10^7^	1.76 × 10^7^	1.47 × 10^6^	8.54 × 10^7^	4.76 × 10^7^	4.27 × 10^7^	5.68 × 10^6^
	Χ^2^	6.60 × 10^−2^	3.65 × 10^−2^	8.17 × 10^−3^	4.80 × 10^−2^	1.20 × 10^−3^	6.10 × 10^−2^	4.30 × 10^−2^	9.90 × 10^−2^
